# Microstructure and Characteristics of Calcium Phosphate Layers on Bioactive Oxide Surfaces of Air-Sintered Titanium Foams after Immersion in Simulated Body Fluid

**DOI:** 10.3390/ma9120956

**Published:** 2016-11-24

**Authors:** Hung-Bin Lee, Hsueh-Chuan Hsu, Shih-Ching Wu, Shih-Kuang Hsu, Peng-Hsiang Wang, Wen-Fu Ho

**Affiliations:** 1 Department of Materials Science and Engineering, Da-Yeh University, Changhua 51591, Taiwan; lhb6018@mail.dyu.edu.tw; 2 Department of Dental Technology and Materials Science, Central Taiwan University of Science and Technology, Taichung 40601, Taiwan; hchsu@ctust.edu.tw (H.-C.H.); scwu@ctust.edu.tw (S.-C.W.); sksheu@ctust.edu.tw (S.-K.H.); 3 Department of Mechanical and Automation Engineering, Da-Yeh University, Changhua 51591, Taiwan; winghsiang@hotmail.com; 4 Department of Chemical and Materials Engineering, National University of Kaohsiung, Kaohsiung 81148, Taiwan

**Keywords:** titanium foam, microstructure, bioactive, hydroxyapatite, simulated body fluid (SBF)

## Abstract

We propose a simple and low-cost process for the preparation of porous Ti foams through a sponge replication method using single-step air sintering at various temperatures. In this study, the apatite-forming ability of air-sintered Ti samples after 21 days of immersion in simulated body fluid (SBF) was investigated. The microstructures of the prepared Ca–P deposits were examined by X-ray diffraction (XRD), field-emission scanning electron microscopy (FE-SEM), Fourier transform infrared (FTIR) spectroscopy, and cross-sectional transmission electron microscopy (TEM). In contrast to the control sample sintered in vacuum, which was found to have the simple hexagonal α-Ti phase, the air-sintered samples contained only the rutile phase. High intensities of XRD peaks for rutile TiO_2_ were obtained with samples sintered at 1000 °C. Moreover, the air-sintered Ti samples had a greater apatite-forming ability than that of the Ti sample sintered in vacuum. Ti samples sintered at 900 and 1000 °C had large aggregated spheroidal particles on their surfaces after immersion in SBF for 21 days. Combined XRD, energy-dispersive X-ray spectroscopy, FTIR spectroscopy, and TEM results suggest that the calcium phosphate deposited on the rutile TiO_2_ surfaces consist of carbonated calcium-deficient hydroxyapatite instead of octacalcium phosphate.

## 1. Introduction

Because of their superior mechanical properties, corrosion resistance, and biocompatibility, titanium (Ti) and its alloys have attracted considerable attention from biomedical researchers [1,2]. Although the use of Ti in orthopedic and dental implants has been successful, clinical problems associated with the mismatch of mechanical modulus between Ti and tissue persist. In particular, the mismatch in the elastic modulus can induce a stress-shielding effect, which eventually leads to excessive bone resorption and artificial loosening of the implant [3]. One feasible way of alleviating this problem is to lower the stiffness of the implant by introducing a porous structure. This minimizes damage to the bone tissue adjacent to the implant and prolongs the device lifetime.

Introducing a porous structure into Ti and its alloys can effectively lower the elastic modulus [4,5]. Finite-element simulations have shown that replacement of Ti with a porous structure may substantially reduce stress-shielding problems [6]. Simultaneously, pores in the implant may promote the entrapment of specific proteins, such as vitronectin and fibronectin, and subsequently enhance the attachment, proliferation, and differentiation of osteoblastic cells on the implant surface [7]. Since the pores provide a surface and space for cell adhesion and bone ingrowth, a direct relationship between pore size and bone formation has been hypothesized [8]. Moreover, the pore interconnection provides a path for cell migration and allows efficient blood vessel formation in vivo [8]. Hsu et al. [9] reported that the inner surfaces of an alkali-treated porous Ti–7.5Mo specimen exhibited a better ability to induce apatite formation. Cell culture results also revealed that the osteoblast-like cell MG63 extends its pseudopodia and attaches well in the pores [9]. Therefore, porous Ti has increasingly been considered as a good candidate for load-bearing bone implants and has been widely studied.

A number of approaches for fabricating porous Ti and Ti alloys have recently been reported, such as slurry foaming [10], gas entrapment technique [11], combustion synthesis [12], and rapid prototyping [13]. Among these methods, rapid prototyping processes based on sintering/melting of powders are capable of rapidly producing parts with complex shapes. For instance, three-dimensional porous Ti–6Al–4V scaffolds have been fabricated by Li’s group by means of a rapid prototyping technology [13]. A new powder metallurgy technique using space holder materials, which offer the advantages of variable porosity, pore shape, and pore size distribution, is in wide use [4,9,14,15]. Hsu et al. [14] prepared porous Ti–7.5Mo alloy scaffolds from ball-milled particles and sintered them at 1100 °C using this method. On the other hand, the sponge replication method can achieve high porosity and good interconnections between pores [16], which benefit bone ingrowth and vascularization of newly formed tissue [7]. Therefore, it is also considered to be a very useful method.

Ti alloys with poor osseointegration of bioinert surfaces in early post-implantation are known to have limited clinical performance and usefulness in broad orthopedic or dental applications. In order to accelerate the healing process and to further improve clinical performance, efforts have been devoted to improving the surface properties of Ti implants [17]. The most commonly used technology in actual clinical application is high-temperature plasma spraying of hydroxyapatite (HA) coating onto Ti alloys [18]. Although this method has produced many good clinical results, it still suffers from problems associated with low fatigue strength, HA degradation, and weak adherence of the coating to the metallic substrate during long-term implantation [19]. To overcome this issue of low bond strength, some other advanced surface coating techniques were developed to compete with the conventional plasma spray techniques, such as cold spraying [20] and laser deposition [21]. Furthermore, applying uniform, thin coatings on implants with complex shapes is difficult [22]. Therefore, different treatments that induce bioactive behavior directly on the metal surface without deposition of a coating have been proposed in the literature. Alkaline treatment using sodium hydroxide (NaOH) is an interesting, simple technique for modifying the Ti implant surface [23]. Recently, Hsu et al. [9] studied apatite formation on porous Ti–7.5Mo alloy treated with a NaOH solution or NaOH + water aging. Nevertheless, the aforementioned processes usually induce surface cracking, result in low fatigue resistance of the treated materials, and are not easily applicable at an industrial scale [24].

According to Wang et al. [25,26] and Feng et al. [27], thermal oxidation can endow Ti with in vitro bioactivity. Other studies have reported that a thick titanium oxide layer can enhance its wear resistance [28], corrosion resistance [29], and biological properties [30]. Thermal oxidation is a relatively simple method for enhancing the surface characteristics and bioactivity of Ti, since it involves increasing the amount of surface hydroxyl groups and the magnitude of the surface energy [27,31]. In our previous study [32], we proposed a low-cost and simple procedure of preparing porous Ti foams by sintering in air. Because of the high melting point of Ti, as well as its high reactivity toward oxygen and impurities at elevated temperatures, fabrication of porous Ti metal is generally difficult. To our knowledge, our method is a novel way of producing Ti foams that uses air sintering instead of sintering in vacuo, as in the conventional method. Moreover, some studies [25,26,27] have indicated that titanium oxide layers on the surface can promote the deposition of apatite when it is immersed in simulated body fluid (SBF). Formation of the apatite layer on the surfaces of bioactive biomaterials in the early stages is considered a key factor for osteoinduction [33]. In our previously published work [32], pore morphology and mechanical properties of the sintered Ti foam have been studied in detail. The proposed novel method can potentially be used in the fabrication of bioactive Ti foams employed as biomaterials. Although the sintering of Ti powder in air could lead to an increase in oxygen content, the present Ti foams exhibit some 3%–14% compressive strain to failure. Therefore, the main goal of the current work is to assess the apatite-forming ability of air-sintered Ti samples after immersion in SBF and to further investigate the microstructure of apatite coatings using X-ray diffraction (XRD), field-emission scanning electron microscopy (FE-SEM), Fourier transform infrared (FTIR) spectroscopy, and cross-sectional transmission electron microscopy (TEM).

## 2. Results and Discussion

### 2.1. Structural Characterization of the Sintered Ti Foam

**Figure 1:**
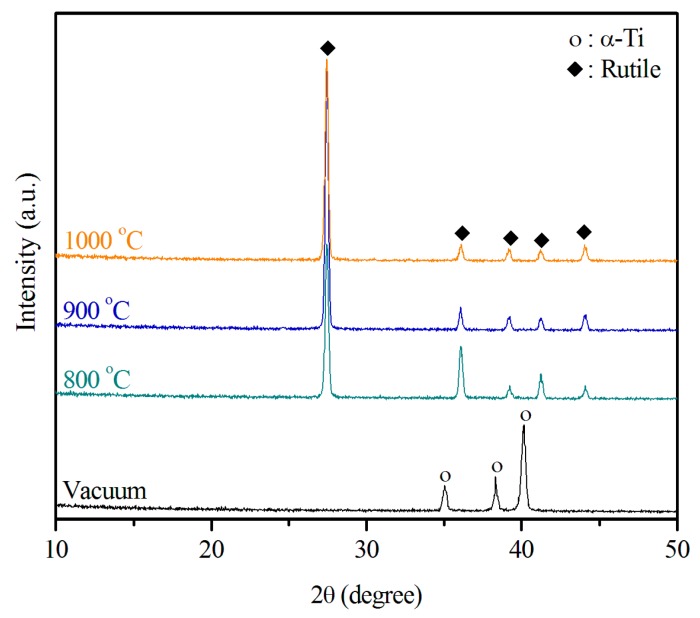
X-ray diffraction (XRD) patterns of Ti foams sintered at 800, 900, and 1000 °C in air and of a foam sintered at 1100 °C in vacuum.

Using the sponge replication method, we fabricated the Ti foams. We used XRD to determine whether any new crystalline phase formed after burnout and sintering at various temperatures. Figure 1 displays the XRD patterns of the samples sintered at 800, 900, and 1000 °C in air, and of the control sample. Clearly, no additional crystalline phase besides the hexagonal α phase formed after treatment of the samples sintered in vacuum. Only the rutile phase could be found in the diffraction patterns of the samples sintered in air. Since the depth of penetration of Cu Kα radiation is in the range of 10–20 μm [34], α-Ti peaks in the XRD patterns of air-sintered Ti samples with a thick oxide layer disappeared. In addition, the samples sintered at 1000 °C produced the most intense rutile TiO_2_ peaks. The amount of rutile phase increased slightly as the temperature increased from 800 to 1000 °C, indicating an increase in the thickness of the titanium oxide layer. The results also reveal that the titanium underwent oxidation during sintering in air and formed films of rutile rather than films of anatase. The rutile structure of titanium oxide, which is more thermally stable than the anatase structure [27], was observed in all samples sintered at the different temperatures.

**Figure 2:**
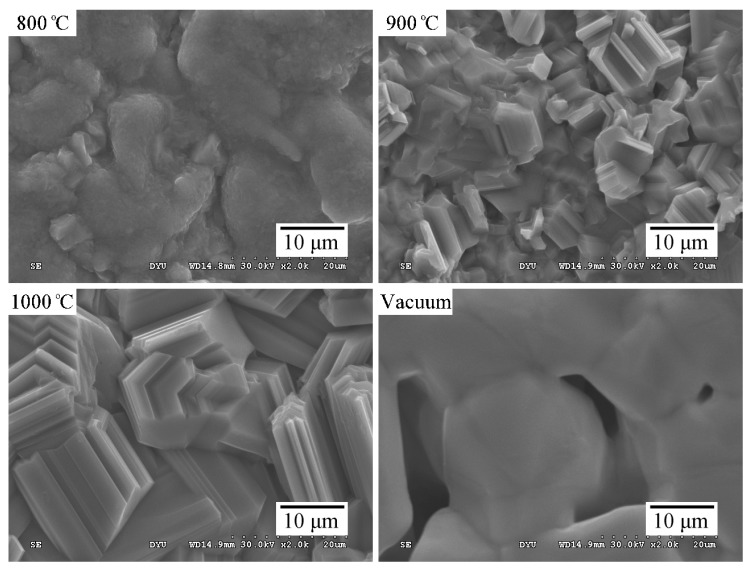
Scanning electron microscopy (SEM) micrographs of the strut surfaces of Ti foams sintered at 800, 900, and 1000 °C in air and of the strut surfaces of a Ti foam sintered at 1100 °C in vacuum.

Figure 2 shows the morphology of the TiO_2_ layers formed on Ti foam air-sintered at 800, 900, and 1000 °C, as well as that of the control sample for comparison. The surfaces noticeably changed as the sintering temperature increased. TiO_2_ particles on the strut surfaces of the Ti foams had a grainy surface structure. They also appeared to be more densely distributed and to have larger crystallites, as shown in the SEM images. In contrast, the control sample exhibited a less dense surface and had micropores with a size of only several microns, which were obtained by the partial sintering of Ti powders. The rough surface microstructure of the sintered strut is another important feature. Because of their large surface area and high wettability, the rough surfaces strongly adsorb biomolecules from biological fluids [35]. Previous studies have found a positive correlation between cell differentiation and surface roughness [34].

### 2.2. Wettability

**Figure 3:**
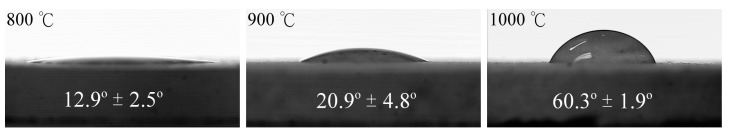
Water contact angles of Ti samples sintered at 800, 900, and 1000 °C in air.

Figure 3 shows the changes in the water contact angles when the samples were air-sintered at 800, 900, or 1000 °C. The samples sintered at 800 °C exhibited a hydrophilic surface with a low contact angle (12.9°). Moreover, the water contact angles of the surfaces increased as the sintering temperature increased. After sintering at 1000 °C, the contact angles increased to around 60.3°. Therefore, the sintering temperature greatly affected the wettability of the oxide layer on the Ti samples. The difference in hydrophilicity among the samples sintered at different temperatures may be explained by the size effect of the TiO_2_ (rutile) crystals. As reported by Park and Kim [36], TiO_2_ films with a smaller grain size have a larger specific surface area; the increased surface area causes the crystal to adsorb more water molecules. The above result is consistent with the SEM results (Figure 2) showing a larger grain size for the samples sintered at a higher temperature.

We confirmed that the surface wettability of an implanted material is an important parameter that influences the biological response to the material. It affects cell behavior in the initial osseointegration process [37], which starts when the implant first contacts blood. Since hydrophilic surfaces facilitate cell proliferation, signals for thrombin and prothrombin activity are predominant and adsorption is stimulated on such surfaces [38]. Furthermore, hydrophilic surfaces stimulate the biomineralization process: Rapid calcium phosphate nucleation proceeds on highly wettable Ti surfaces [39]. On the other hand, hydrophobicity is not conducive to protein adsorption [40]. Therefore, the appropriate surface wettability is necessary for coatings. Webb et al. showed that hydrophilic surfaces with water contact angles of 20°–40° promote the highest levels of cell attachment [41]. As a result, the sample sintered at 900 °C in the present study appeared to have the appropriate surface wettability, which corresponds to a contact angle of 20.9°.

### 2.3. Ca–P Precipitation on the Sintered Surfaces

**Figure 4:**
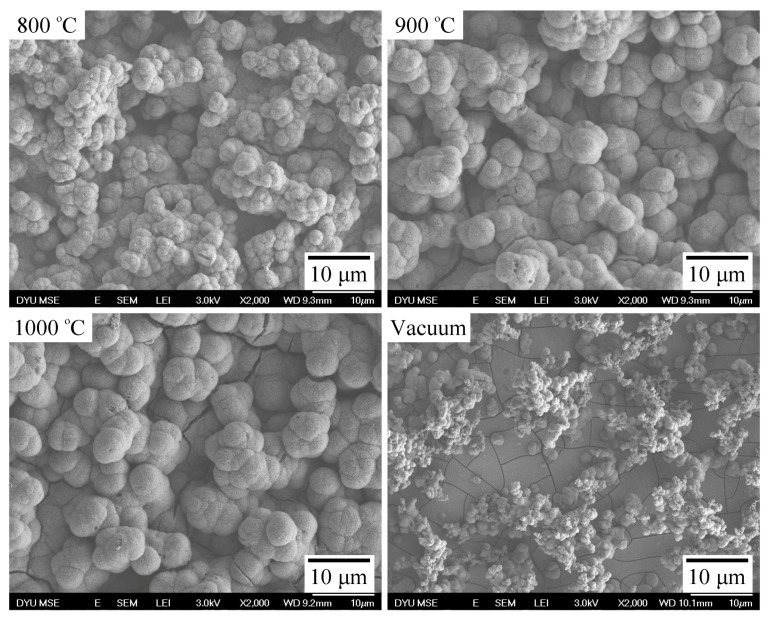
SEM micrographs of the surface morphology of Ti samples sintered at 800, 900, and 1000 °C in air and of a Ti sample sintered in vacuum after immersion in simulated body fluid (SBF) for 21 days.

Figure 4 shows the surface SEM photographs of the air-sintered Ti samples that were immersed in SBF for 21 days. The sample sintered in vacuum was used as a control. Apatite formed on the surfaces of all sintered Ti samples after immersion. Numerous spherulites covered all surfaces of the three samples sintered in air, suggesting good bioactivity of the samples. However, only numerous smaller apatite nucleation spheres and deposits were present on the surfaces of the Ti sample sintered in vacuum. Notably, the ability of this Ti sample to form apatite was markedly lower than that of its air-sintered counterparts. Furthermore, the aggregated island-like spheroidal particles on the surfaces of Ti samples sintered at 900 and 1000 °C were much larger than those of the spheroidal particles on the samples sintered at 800 °C.

**Figure 5:**
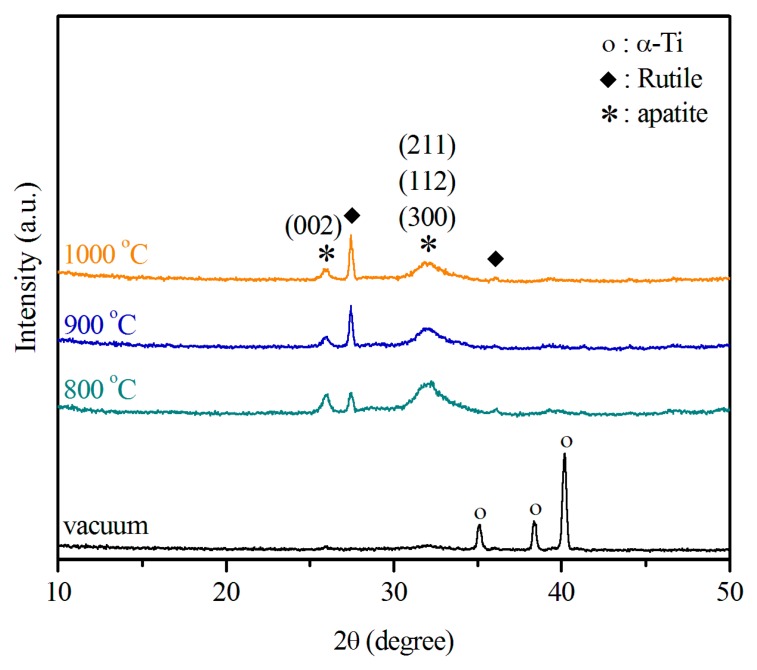
XRD patterns of Ti samples sintered at 800, 900, and 1000 °C in air and of a Ti sample sintered in vacuum after immersion in SBF for 21 days.

XRD patterns of the sintered Ti samples immersed in SBF for 21 days (Figure 5) contain peaks for apatite, which are almost negligible in the pattern for the sample sintered in vacuum. The peak at 2θ of 31.9° corresponds to the overlapping (211), (112), and (300) diffraction planes, and that at 2θ of 25.9° is due to the (002) diffraction plane. Both peaks match those of the hydroxyapatite (HA) phase. The apatite phase produced broad diffraction peaks, suggesting an HA phase with low crystallinity and tiny crystallites. Diffraction peaks produced by the Ti substrate disappeared from the XRD patterns on the oxidized Ti substrate, indicating that the apatite layer that formed on its surface thickened, thereby suppressing diffraction by the Ti substrate. On the contrary, the diffraction peaks of Ti could still observed in the pattern of the sample sintered in vacuum, whereas those of apatite could be barely observed.

**Figure 6:**
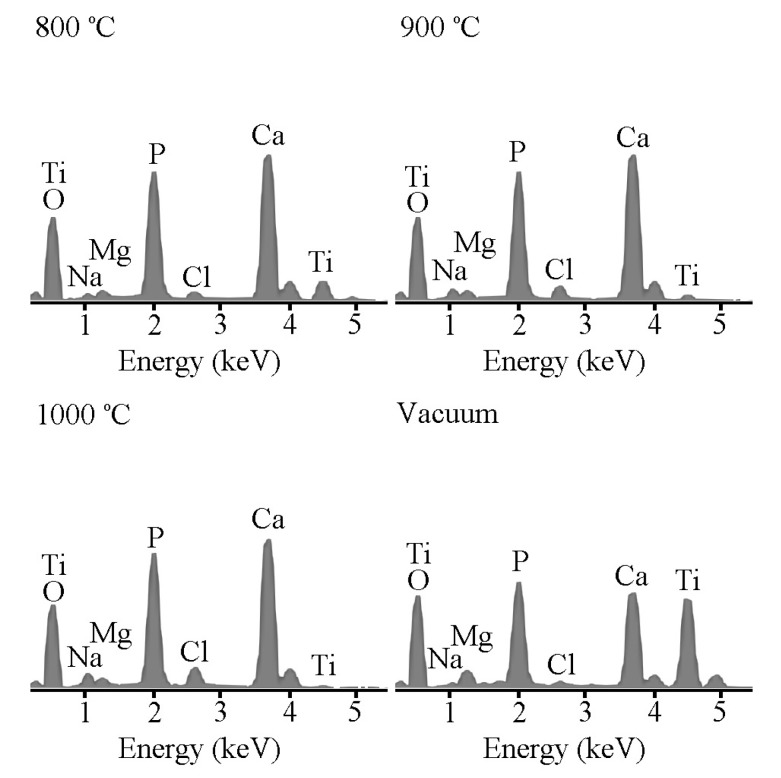
Energy-dispersive X-ray spectroscopy (EDS) results for Ti samples sintered at 800, 900, and 1000 °C in air and for a Ti sample sintered in vacuum after immersion in SBF for 21 days.

Energy-dispersive X-ray spectroscopy (EDS) analysis of all sintered Ti samples revealed Ca, P, Na, Mg, Cl, O, and Ti after 21 days of immersion in the SBF (Figure 6). The Ti samples air-sintered at 800, 900, and 1000 °C produced very intense peaks for Ca and P and a relatively weak peak for Ti. The intensity of the peaks for the substrate Ti drastically decreased because of interference from the Ca–P deposits after immersion in SBF. However, the Ti sample sintered in vacuum produced Ca and P peaks with intensities lower than those of peaks for the air-sintered samples; in contrast, its Ti peak is more intense than the Ti peaks for the air-sintered samples. These results indicate that the surfaces of the three air-sintered Ti samples were completely covered with apatite. The ability of the Ti sample sintered in vacuum to form apatite was apparently lower than that of its counterparts sintered in air. Furthermore, a longer immersion time and continuous mineral deposition changed the Ca/P molar ratios of the four samples. After immersion in SBF for 21 days, the Ca/P molar ratio in the sample sintered in vacuum was only 1.20 ± 0.03; whereas it reached 1.43 ± 0.04, 1.44 ± 0.02, and 1.41 ± 0.02 for the samples air-sintered at 800, 900, and 1000 °C, respectively. These Ca/P molar ratios are smaller than the ratio of 1.67 for stoichiometric HA, thus implying that the final biomineralization products were calcium-deficient. Furthermore, the three air-sintered Ti samples had high Ca/P atomic ratios (1.41–1.44), which fall between those of octacalcium phosphate (OCP; Ca/P atomic ratio = 1.33) and calcium-deficient HA (CDHA; Ca/P atomic ratio = 1.5). Several studies have reported that biomimetic apatites have relatively low Ca/P ratios because of the small amounts of sodium (Na^+^), magnesium (Mg^2+^), chloride (Cl^−^), and carbonate (CO_3_^2−^) present in their lattice structure [42].

**Figure 7:**
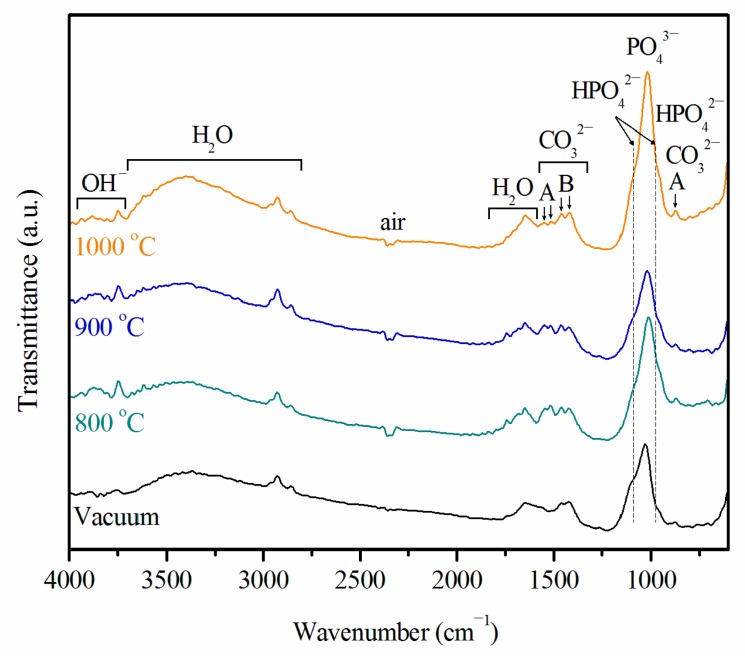
Fourier transform infrared (FTIR) spectra of Ti samples sintered at 800, 900, and 1000 °C in air and of a Ti sample sintered in vacuum after immersion in SBF for 21 days. A: A-type carbonate; B: B-type carbonate.

FTIR spectra of all sintered Ti samples that were immersed in SBF for 21 days are displayed in Figure 7. The four samples have similar FTIR spectra, indicating that their Ca–P coating layers had the same functional groups. Bands assignable to OH^−^, PO_4_^3−^, CO_3_^2−^, HPO_4_^2−^, and H_2_O are present. These results suggest that all of the coating layers have carbonate-substituted calcium phosphates. In the present study, the peaks at 1421 and 1459 cm^−1^ are characteristic of B-type carbonate-containing HA, while the peaks at 867, 1515, and 1545 cm^−1^ are characteristic of A-type carbonate. FTIR peaks at 1019 cm^−1^ are due to PO_4_^3−^. The HPO_4_^2−^ bands at 867, 963, and 1090 cm^−1^ could be due to the OCP or CDHA phase. The O–H group at 3700−3750 cm^−1^ confirms that the coatings are related to the HA structure. On the basis of the XRD, EDS, and FTIR results, the coating structure may be carbonated CDHA, which is similar to the mineral component of bone.

A biomimetic coating technique using SBF was adopted to activate the Ti metal. The SBF had ion concentration, temperature, and pH very similar to those encountered in physiological conditions. Some researchers have reported that bone-like HA forms the biomimetic coating [43,44]. SBF can induce deposition of CDHA coatings similar in composition to the mineral component of bone on suitably treated Ti surfaces [33,45]. However, other researchers maintain that the coating could be made up of the OCP phase. For example, Lu and Leng investigated the formation of calcium phosphate on NaOH-treated Ti surfaces using TEM [46]. They proposed that OCP instead of HA directly nucleates from amorphous calcium phosphate and continuously grows on NaOH-treated Ti surfaces rather than transforming into apatite. Yang et al. [47] confirmed OCP precipitates from the main peaks of OCP (002) and OCP (402) in their XRD spectrum. Notably, standard powder XRD patterns of HA and OCP are similar in the 20°–40° 2θ range. These two peaks may also be assigned to CDHA (002) and CDHA (211), as shown in Figure 5. The composition and structure of the Ca–P coating can be influenced by factors such as SBF composition and surface treatment conditions. Although we infer from our XRD, EDS, Ca/P ratio, and FTIR results that the coating layer is poorly crystalline carbonated CDHA, further examination of the structure of the Ca–P coating is important. To our knowledge, no study has investigated the structure and composition of this coating on the rutile surfaces of air-sintered Ti samples that have been immersed in SBF.

**Figure 8:**
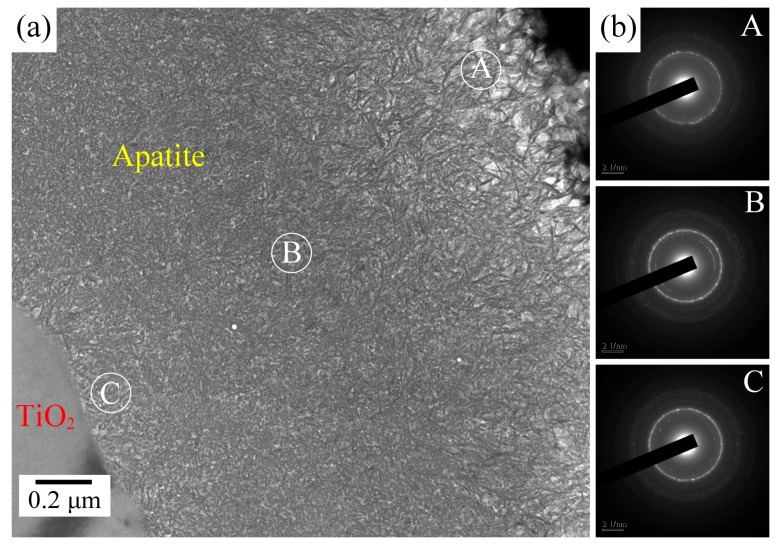
(**a**) Cross-sectional transmission electron microscopy (TEM) image of the Ca–P layer of a Ti sample air-sintered at 900 °C after 21 days of immersion in SBF; (**b**) Three selected-area electron diffraction (SAED) patterns taken from different spots (A, B, and C) indicated in the image of the apatite layer.

The coating microstructure of the sample air-sintered at 900 °C after 21 days of immersion in SBF was investigated further by cross-sectional TEM. The TEM image in Figure 8a shows a cross-sectional view of the apatite layer above the titanium oxide layer. We found the coating to be about 2 μm thick. The top layer of the coating was a loose and porous structure, whereas the bottom layer exhibited a more dense structure. A biomimetic apatite coating grew on the surface of titanium oxide by the attachment of Ca^2+^ and PO_4_^3−^ ions from SBF. The corresponding selected-area electron diffraction (SAED) patterns, taken from three spots on the apatite coating (spots “A”, “B”, and “C”, which are respectively located on top, in the middle, and at the bottom of the coating), are shown in Figure 8b. The three spots have the same diffraction patterns, thus indicating that the Ca–P coating has the same structure from the bottom to the top regions.

**Figure 9:**
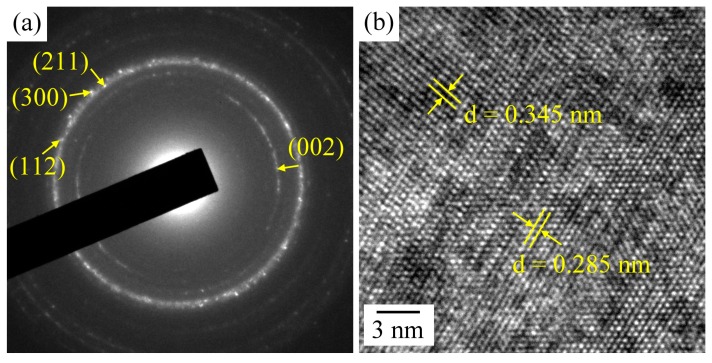
(**a**) SAED pattern taken from the middle area of the Ca–P layer (spot B in Figure 8a); (**b**) high-resolution TEM (HRTEM) image of the Ca–P layer.

In the present study, SAED was used to identify a nanometer-sized area of coating to obtain local crystallographic information with high d-spacing accuracy. The SAED pattern taken from spot B of Figure 8a (Figure 9a) exhibits a number of rings typical of poorly crystalline and nanocrystalline structures. All distances correspond exactly to the hexagonal structure of HA. The crystal structure of the deposited Ca–P coating was further evaluated by high-resolution TEM (HRTEM). Well-defined lattice fringes with *d*-spacings of 0.345 and 0.285 nm are distinct, as shown in Figure 9b. On the basis of the SAED and HRTEM images, these distances can be assigned to the (002) and (211) lattice planes of HA (Joint Committee for Powder Diffraction Studies (JCPDS) File No. 86-1203). Additionally, the (211), (112), and (300) diffraction planes of CDHA can be indexed to the SAED pattern of Figure 9a because of their very similar interplanar distances (0.28 nm), which are in agreement with the XRD result (Figure 5). Furthermore, OCP was not present in the Ca–P coating, as evidenced by the absence of diffraction rings corresponding to *d*-spacings of 0.938, 0.932, and 0.683 nm, which are characteristic of OCP [48]. Therefore, the above results confirm the presence of the Ca–P coating layer with carbonated CDHA instead of a layer with OCP.

## 3. Materials and Methods

### 3.1. Ca–P Precipitation on the Sintered Surfaces

**Figure 10:**
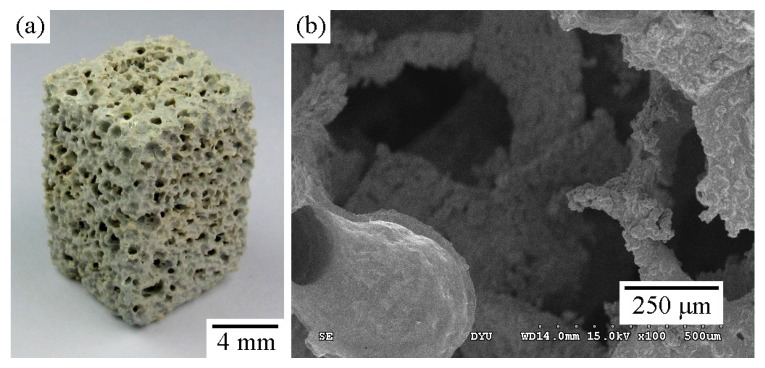
Photograph of a Ti foam air-sintered at 800 °C. The typical sample of the Ti foam (**a**), and SEM micrograph of the porous Ti (**b**).

The porous Ti scaffold was fabricated through a polymer replication method, which has been reported in detail [32]. In brief, samples were heated at a rate of 2 °C/min to 400 °C, which was subsequently held for 2 h to burn out the polyurethane (PU) sponges. The samples were then heated at a rate of 20 °C/min to specified temperatures (800, 900, or 1000 °C), which were subsequently held for 1 h. All heat treatments were conducted in air in an electric furnace. Samples used for comparison were prepared by sintering in vacuum at 1100 °C for 15 h at a heating rate of 10 °C/min. Figure 10 shows a typical Ti foam sintered at 800 °C for 1 h. Statistical analysis revealed that >90% of the pores were larger than 100 μm and that the total porosities of the Ti foams sintered were >79%. Morphological examination showed that most pores were interconnected through channels.

### 3.2. Examination of Apatite-Forming Ability

The surface wettability of the sintered Ti foam specimen was evaluated by contact angle measurements. Experiments were carried out using highly pure water, which was placed on each sample by means of a micropipette. A charge-coupled-device (CCD) camera was used to photograph the shape of the drops, and the contact angle in the image was measured. Only non-porous specimens were used in this study. The contact angle for each material type and the treatment conditions were reported as means of six measurements.

The in vitro bioactivity of all samples was evaluated in terms of their apatite-forming ability during immersion in SBF. We prepared the SBF by dissolving reagent-grade NaCl, NaHCO_3_, KCl, K_2_HPO_4_·3H_2_O, MgCl_2_·6H_2_O, CaCl_2_, and Na_2_SO_4_ in distilled water, adjusting the pH of the resulting solution to 7.4 with Tris and HCl [49]. Ionic concentrations in the SBF and in human blood plasma are listed in Table 1. Ti foam sintered in vacuum at 1100 °C for 15 h was used as a control. All Ti foam samples were sequentially washed with acetone and ethanol each for 20 min. The samples were then washed in distilled water for an additional 10 min and immersed in 15 mL of SBF at 37 °C for 21 days. The SBF was replenished every 2 days to preserve its ion concentration. The temperature was maintained using a water bath. After immersion for a predetermined period, the specimens were removed from the SBF, gently washed with distilled water, and air-dried. The surface morphology of apatite formed on each specimen was examined by FE-SEM (JSM-6700F, JEOL, Tokyo, Japan). The crystallinity of the phases formed on the surfaces of the samples was measured by high-resolution XRD (D8 SSS, Bruker AXS, Madison, WI, USA). Surface chemical analysis was performed by energy-dispersive X-ray spectroscopy (EDS) coupled with SEM (INCA Energy 350, Oxford Instruments, Oxford, UK). FTIR reflection spectroscopy (IRAffinity-1; Shimadzu, Kyoto, Japan) was performed on the specimen surface. To examine cross-sectional microstructures and lattice structures of the biomimetic coating, the samples were prepared by using a focused ion beam (JIB-4601F, JEOL, Tokyo, Japan) and then examined by TEM (JEM-2100F, JEOL, Tokyo, Japan).

## 4. Conclusions

In the present study, Ti foams that had a bioactive oxide layer were fabricated by single-step sintering in air at three heating temperatures. Their apatite-forming ability was examined by immersing them in SBF for 21 days. Then the microstructure of the apatite deposits on the foams was investigated. The results obtained in this research are summarized as follows:

Only the rutile phase was found in the XRD patterns for the air-sintered samples, whereas only the hexagonal α-Ti phase was identified in the XRD patterns for the sample sintered in vacuum. The samples sintered at 1000 °C produced the most intense rutile TiO_2_ peaks.

The water contact angles on the surfaces of the air-sintered samples increased as the sintering temperature increased from 800 to 1000 °C. The sample sintered at 900 °C exhibited an appropriate surface wettability, which corresponded to a contact angle of 20.9°.

The apatite-forming ability of the air-sintered Ti samples was greater than that of the Ti sample sintered in vacuum. Large spheroidal particles aggregated on the surfaces of Ti samples sintered at 900 and 1000 °C.

Apatite that formed on the surfaces of the sintered Ti samples had relatively low Ca/P ratios because of the small amounts of sodium, magnesium, chloride, and carbonate present in their lattice structure.

FTIR results showed that the Ca–P coating layer contains carbonate; specifically, A- and B-type carbonated apatite. Furthermore, SAED and HRTEM images confirmed the formation of a Ca–P coating with carbonated CDHA instead of a coating with OCP.

## Figures and Tables

**Table 1:** Ionic concentrations (mM) in simulated body fluid (SBF) and in human blood plasma [49].

Ion concentration (mM)	Na^+^	K^+^	Mg^2+^	Ca^2+^	Cl^−^	HPO_4_^2−^	SO_4_^2−^	HCO_3_^−^
Blood plasma	142.0	5.0	1.5	2.5	103.0	1.0	0.5	27.0
SBF	142.0	5.0	1.5	2.5	147.8	1.0	0.5	4.2
